# Comparative Assessment of Smile Attractiveness Perception and Treatment Need Across Dental Professionals and Laypersons

**DOI:** 10.7759/cureus.94037

**Published:** 2025-10-07

**Authors:** Shalini Ravichandra, Khadeer Riyaz, Laxmikanth S Manjappa, Ashita Talwar, Nandan K Narsimha Mogaveera, Partha P Ghosh, Seema Gupta

**Affiliations:** 1 Department of Orthodontics, The Oxford Dental College, Bengaluru, IND; 2 Department of Orthodontics, Kothiwal Dental College and Research Centre, Moradabad, IND

**Keywords:** esthetics, laypersons, orthodontist, perception, smile

## Abstract

Introduction: Smile esthetics are crucial in orthodontics; however, orthodontists, general dentists, and laypersons perceive deviations differently, prompting the need for comparative evaluation. This study explored how these groups assess upper midline discrepancies, gingival display, maxillary cant, reduced upper teeth show, asymmetric upper lip elevation, and chin deviation, aiming to determine their combined impact on smile attractiveness and treatment needs while identifying thresholds for clinical intervention to enhance patient-focused care.

Methodology: This cross-sectional study was conducted in the Department of Orthodontics from August 2023 to October 2024. A frontal smile photograph was digitally manipulated to create six series of images (54 images), each with nine variations of upper midline displacement with respect to facial midline (-2 to +2 mm in 0.5 mm increments). Modifications included increased gingival exposure (gumminess), a 5° maxillary cant, reduced upper teeth show, 2 mm asymmetric upper lip elevation, and 2 mm chin deviation. Ninety evaluators (30 orthodontists, 30 general dentists, 30 laypersons) assessed the images using a visual analog scale (VAS) for attractiveness and indicated the need for treatment. Data were analyzed using one-way analysis of variance (ANOVA) for VAS scores and chi-square tests for treatment need, with intra-evaluator reliability assessed using intraclass correlation coefficients.

Results: Orthodontists demonstrated significantly higher sensitivity to minor upper midline deviations (-1.0 mm to 0.0 mm), maxillary cant, and asymmetric upper lip elevation compared to general dentists and laypersons (*P* < 0.05), with significant intergroup differences in perceived treatment need for all conditions except chin deviation at the ideal midline (0.0 mm). As deviations increased beyond 0.5 mm, perceptions converged, becoming statistically indistinguishable (*P* > 0.05). Orthodontists recommended treatment most frequently (>90% for 1.5-2.0 mm deviations), particularly for chin deviation and upper lip asymmetry, while laypersons showed the lowest treatment need perception (40%-60% *No* for minor deviations). Increased gingival display and chin deviation amplified the treatment need across groups, with same-side chin and midline deviations less noticeable than opposite-side deviations.

Conclusions: Professional expertise significantly influenced the detection of subtle smile deviations, with orthodontists advocating treatment more often than laypersons. These findings emphasize the need for tailored patient communication to align clinical interventions with patient expectations and enhance patient-centered orthodontic outcomes.

## Introduction

Today, the paradigm has shifted toward enhancing overall facial esthetics, with smiles being a key determinant of treatment success [1]. A harmonious smile, defined by Webster as a facial expression involving brightened eyes and an upward curve of the mouth, conveys emotions ranging from pleasure to approval, and significantly influences social perceptions [1,2]. For young adults, the esthetic outcome of orthodontic treatment, particularly the smile and soft tissue profile, is often a critical factor in their satisfaction [3].

The components of an attractive smile include the lips, gingival contours, buccal corridors, tooth proportions, and alignment of the dental midline with the facial midline [4,5]. Factors such as gingival display, midline discrepancies, and chin deviation can disrupt smile harmony [5]. Excessive gingival exposure, often termed a *gummy smile*, has been studied extensively, with esthetic thresholds ranging from 1 to 3 mm of gum visibility [6,7]. Treatment options for excessive gingival display include periodontal procedures, orthodontic interventions, or surgical approaches, although achieving symmetry remains challenging [8]. Similarly, chin deviation, often resulting from skeletal asymmetries, congenital deformities, or functional shifts, can exacerbate facial asymmetry, impacting both esthetics and function [9]. Upper dental midline discrepancies, when combined with these factors, further complicate achieving an ideal smile [10].

Despite extensive research on individual smile components, the combined effects of upper midline discrepancies, gingival display, and chin deviation on smile attractiveness remain underexplored. Understanding how these factors are perceived by orthodontists, general dentists, and laypersons is crucial, as their perspectives often differ owing to varying levels of training and esthetic sensitivity. Therefore, this study aimed to compare the perceptions of orthodontists, general dentists, and laypersons in detecting deviations from ideal smile esthetics, particularly in the context of upper midline discrepancies, gingival display, and chin deviation. The objective of this study was to evaluate the influence of upper midline discrepancies combined with gingival display, reduced upper teeth show, chin deviation, maxillary cant, and asymmetric upper lip elevation on smile attractiveness. By identifying the thresholds at which these deviations become noticeable, this study seeks to inform clinical decision-making and enhance patient-centered orthodontic outcomes.

## Materials and methods

This cross-sectional perceptual study was conducted in the Department of Orthodontics at The Oxford Dental College, Bengaluru, Karnataka, India, from August 2023 to October 2024. Ethical approval was obtained from the Institutional Review Board of The Oxford Dental College (Approval No. TODC/018/ECAL/2022-23, May 5, 2023), and the study adhered to the principles of the Declaration of Helsinki. Written informed consent was obtained from all the evaluators before participation.

The sample size was calculated using G*Power software (version 3.1.9.7; Heinrich-Heine-Universität Düsseldorf, Düsseldorf, Germany) based on a previous study by Najafi et al. [11], which compared aesthetic ratings of images among orthodontists, prosthodontists, and laypersons, the analysis indicated a minimum requirement of 87 participants with an effect size of 0.34, an alpha error of 5% (α = 0.05), and a power of 80%. The calculation was rounded to 90 for practical purposes, divided into three groups of 30 each: orthodontists, general dentists, and laypersons.

A total of 90 evaluators participated in this study, including 30 orthodontists, 30 general dentists, and 30 laypersons. The mean ages of the layperson, general dentist, and orthodontist groups were 32 ± 9.53, 34 ± 7.81, and 36 ± 8.62 years, respectively. There was equal sex representation in all the groups. The orthodontists and general dentists (with a degree of Bachelor's of Dental Surgery) had an average professional experience of more than eight years. Laypersons were recruited from the local community, and those with dental experience were excluded. Most laypersons were college-educated.

The pre-existing standardized frontal smile image with the chin area of a female model (aged 25 years) with ideal facial esthetics served as an ideal template for the study. This image served as the control (Series A) and was digitally manipulated using Adobe Photoshop Creative Suite (version 8.0, Adobe Inc., San Jose, CA) to create five additional series (B, C, D, E, and F), each with nine images in which the upper dental midline was displaced horizontally from -2 mm (left) to +2 mm (right) in 0.5 mm increments with respect to facial midline, resulting in 54 images. The modifications were designed to simulate common variations observed in smile esthetics and were verified by an experienced orthodontist for clinical relevance.

**Figure 1:**
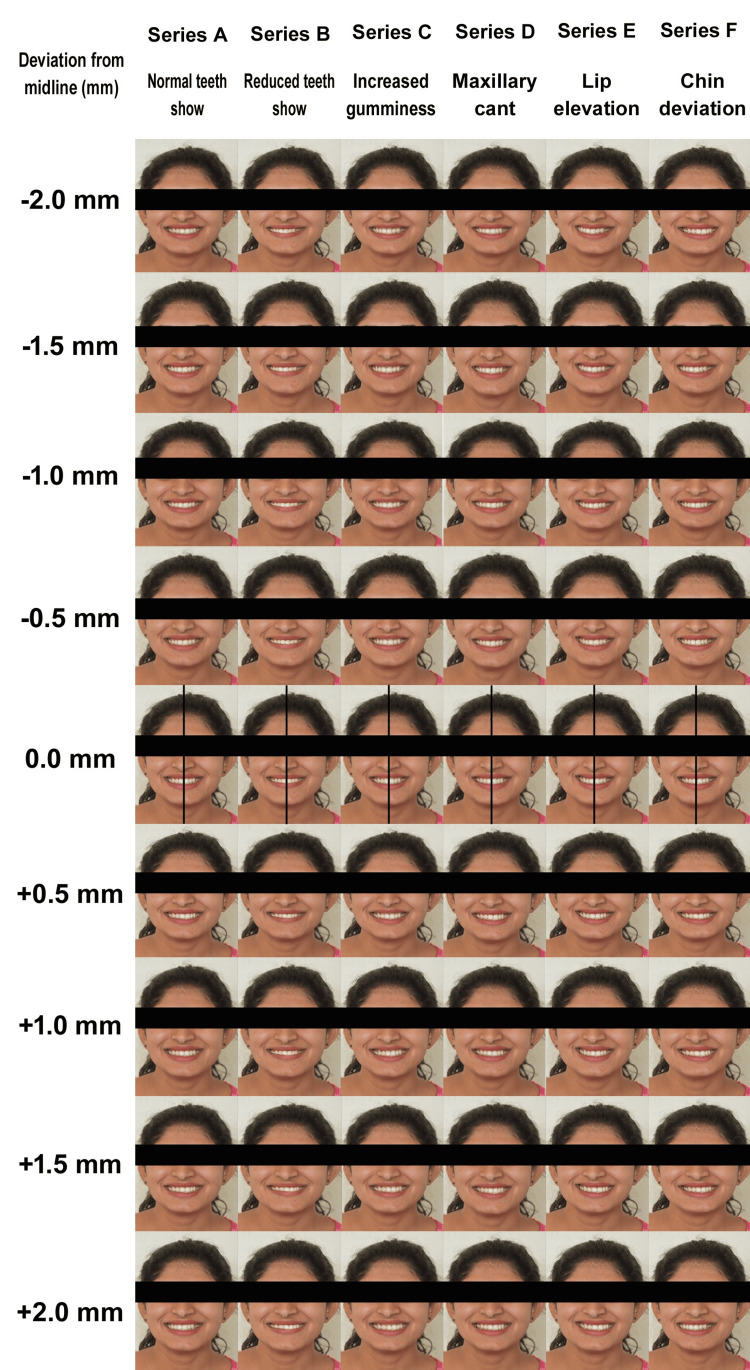
Series of edited photographs for perception.

The series were defined as follows: Series A (control) showed the ideal smile with full upper anterior crown display and no gingival exposure; Series B (decreased upper teeth show) had gingival show symmetrically reduced by 2 mm; Series C (increased gumminess) had gingival show symmetrically increased to a maximum of 3 mm at the upper central incisor area; Series D (maxillary cant) involved a 5° rotation of the upper dentition around the incisal contact point of central incisors, with the right side shifted downward and the left side elevated; Series E (asymmetric upper lip elevation) had the right commissure elevated by 2 mm; and Series F (chin deviation) featured a 2 mm chin midline deviation to the right and left sides. Image processing was performed using a Dell Inspiron 15 7000 Series computer (Dell Technologies, Round Rock, TX) (Figure 1; Appendix).

A calibration session, conducted by a single trained observer, instructed evaluators on using a Visual Analog Scale (VAS) from 1 (least attractive) to 100 (most attractive), assessing treatment need (*Yes* or *No*), and familiarizing them with the evaluation process through a practice session with sample images. The 54 photographs were divided into six series (A-F), each with nine images randomized using Microsoft Excel (Microsoft Corporation, Redmond, WA) to minimize the order bias. In the evaluation process, raters assessed each photograph for both smile attractiveness and perceived treatment need. *Treatment need* was defined as the evaluator’s judgment on whether orthodontic intervention was required to address the observed smile deviations, based on their professional expertise (for orthodontists and general dentists) or personal aesthetic perception (for laypeople). For each image, raters provided a binary response (Yes/No) to the question: Does this smile require orthodontic treatment to improve its aesthetics? This response was recorded alongside the VAS score for attractiveness on the provided questionnaire. To ensure clarity, raters were instructed during the calibration session that *treatment need* specifically referred to the necessity of orthodontic correction to achieve an aesthetically acceptable smile, considering the specific deviations presented (e.g., midline displacement, gingival display, maxillary cant, asymmetric lip elevation, or chin deviation).

The sets of photographs were arranged according to their respective groups; however, within each set, the images displaying incremental changes were randomized to minimize the order bias. Questionnaires were then distributed to all the evaluators. Each questionnaire consisted of two parts: the first section elicited demographic information, including nationality, sex, and age, while the second section contained a VAS for rating the photographs. The evaluators were instructed to score the attractiveness of each image separately on a VAS ranging from least to most attractive. To ensure consistency and avoid prolonged deliberation, the time allotted for the evaluation of each image was limited to one minute. They independently scored smile attractiveness and indicated the need for treatment in a controlled environment with consistent lighting.

For intra-evaluator reliability, 30% of the evaluators from each group (nine orthodontists, nine general dentists, and nine laypersons) re-evaluated the randomized image sets and questionnaires two weeks after the initial assessment. Intraclass correlation coefficients (ICC) were calculated to assess the consistency of the VAS scores and treatment need responses, yielding high reliability with ICC values ranging from 0.85 to 0.92 for VAS scores and 0.80 to 0.89 for treatment need assessments across all groups, indicating strong consistency in the evaluations of the groups.

Data were analyzed using the Statistical Package for the Social Sciences (SPSS) software (version 26; IBM Corp., Armonk, NY). The Shapiro-Wilk test confirmed the normality of the VAS scores, permitting the use of parametric one-way analysis of variance (ANOVA) to compare the mean ratings between the participant groups. Continuous data are presented as mean ± standard deviation, and categorical data as frequency and percentage. The association between the groups and treatment needs was assessed using the chi-square test, with a *P*-value < 0.05 considered statistically significant.

## Results

The descriptive analysis of VAS scores for perceived treatment need revealed a clear trend across all evaluators (general dentists, laypersons, and orthodontists). As the midline deviation increased from -2 to 2 mm, the mean VAS scores decreased for most altered conditions, including reduced upper teeth show, increased gumminess, maxillary cant, asymmetric upper lip elevation, and chin deviation. This indicates that larger deviations are perceived as less in need of correction, suggesting potential adaptation or acceptance of more significant asymmetries. Orthodontists consistently reported the highest mean VAS scores at minor deviations (-0.5 to 0 mm), indicating a higher sensitivity and perceived treatment need for subtle discrepancies compared to other groups. Conversely, all groups showed the lowest scores for major deviations (1.5 mm, 2 mm), particularly for chin deviation. The inference is that the perception of treatment needs is highly dependent on the magnitude of the deviation (Table 1).

**Table 1:** Descriptive analysis of visual analog scale (VAS) scores for smile attractiveness in altered smile conditions with different midline deviations, as evaluated by general dentists, laypersons, and orthodontists. VAS scores are presented as mean and standard deviation (SD).

Deviation of upper midline with respect to facial midline (mm)	Evaluators	Series A: Normal upper teeth show		Series B: Reduced teeth show		Series C: Increased gumminess		Series D: Maxillary cant		Series E: Asymmetric upper lip elevation		Series F: Chin deviation	
		Mean	SD	Mean	SD	Mean	SD	Mean	SD	Mean	SD	Mean	SD
-2	General dentists	64.33	14.55	59.00	16.68	63.33	11.55	61.00	11.55	48.67	11.67	53.00	12.08
	Laypersons	63.33	18.82	64.00	14.76	64.33	16.54	65.67	16.75	54.00	17.34	56.67	17.09
	Orthodontists	67.33	18.18	67.00	16.85	60.67	12.85	64.33	16.33	60.37	20.15	60.37	18.92
-1.5	General dentists	65.67	16.75	60.33	14.74	62.00	10.31	63.33	13.98	51.67	11.47	55.67	10.40
	Laypersons	64.00	16.10	61.67	13.92	63.67	16.91	69.00	16.47	57.00	15.35	57.33	17.60
	Orthodontists	68.00	16.27	64.33	18.70	63.33	12.69	66.67	15.83	62.67	17.99	58.03	17.39
-1	General dentists	64.00	13.54	61.00	11.55	62.00	14.48	60.00	13.13	53.33	11.55	59.33	13.88
	Laypersons	64.00	14.99	66.67	12.13	66.00	16.10	68.67	16.34	63.00	17.05	58.33	17.24
	Orthodontists	71.00	15.61	64.00	16.53	70.00	12.32	70.33	12.17	66.33	12.99	64.00	14.53
-0.5	General dentists	65.00	18.34	62.33	14.31	64.33	13.05	61.33	14.79	55.67	11.65	60.33	14.26
	Laypersons	67.00	18.41	69.33	12.58	68.33	18.77	67.67	15.47	64.33	17.56	65.33	18.14
	Orthodontists	73.33	14.46	70.67	18.37	73.67	11.89	73.67	15.20	69.67	14.02	69.00	16.26
0	General dentists	64.00	13.03	61.00	19.54	61.00	13.22	61.33	15.03	57.33	14.84	65.67	14.55
	Laypersons	66.67	17.88	66.33	16.50	63.33	21.55	65.67	15.01	64.67	16.76	69.00	16.47
	Orthodontists	74.67	11.37	75.33	15.03	72.33	13.05	74.00	11.63	69.67	15.86	69.00	17.29
0.5	General dentists	60.00	16.61	59.33	16.39	59.00	12.69	61.33	14.56	59.33	13.11	58.00	15.18
	Laypersons	62.33	19.77	63.00	17.45	63.00	22.00	64.00	17.14	61.67	16.21	67.33	17.60
	Orthodontists	67.67	13.05	66.33	14.74	68.00	13.24	67.70	17.02	67.67	16.33	64.00	16.10
1	General dentists	59.67	17.91	59.00	13.22	61.33	14.08	59.00	15.17	59.00	17.09	56.00	13.54
	Laypersons	59.53	20.43	58.00	17.50	61.33	22.85	64.33	18.32	61.33	16.13	62.67	20.33
	Orthodontists	61.67	14.16	62.67	17.01	63.00	13.43	66.70	16.75	61.67	15.99	59.03	17.77
1.5	General dentists	60.67	17.99	58.67	14.08	60.33	14.02	61.00	15.17	54.00	15.22	52.67	16.39
	Laypersons	57.33	18.18	56.00	20.10	59.67	20.59	63.00	20.37	58.67	16.13	57.00	18.41
	Orthodontists	57.67	12.78	60.70	17.29	58.70	17.84	65.37	14.90	58.70	18.97	56.37	17.99
2	General dentists	63.00	16.85	55.67	16.33	58.00	16.69	62.67	14.61	57.00	14.42	53.67	17.52
	Laypersons	61.33	20.80	57.67	22.39	57.00	24.09	67.33	17.60	58.67	21.29	53.33	21.23
	Orthodontists	56.67	16.88	59.37	18.45	55.70	15.79	60.70	18.07	56.03	19.84	52.03	19.98

The one-way ANOVA results revealed that statistically significant differences (*P* < 0.05) in perceived treatment need between evaluators were almost exclusively concentrated at minor midline deviations, particularly from -1.0 to 0.0 mm. For the 0.0 mm (ideal) midline, significant intergroup differences existed for all conditions, except chin deviation. As the magnitude of deviation increased beyond 0.5 mm, the *P*-values became non-significant across all altered conditions. This indicates that while the three groups disagree on the treatment need for very subtle or ideal esthetics, their perceptions converge and become statistically indistinguishable when presented with more pronounced, clinically obvious asymmetries of 1.0 mm or greater. Professional training and expertise significantly influenced the sensitivity to minor discrepancies (Table 2).

**Table 2:** Comparative analysis of mean visual analog scale (VAS) scores for smile attractiveness in altered smile conditions with different midline deviations between evaluators. *P < 0.05 denotes statistical significance using the one-way analysis of variance (ANOVA) test.

Deviation of upper midline with respect to facial midline (mm)	Series A: Normal upper teeth show		Series B: Reduced upper teeth show		Series C: Increased gumminess		Series D: Maxillary cant		Series E: Asymmetric upper lip elevation		Series F: Chin deviation	
	*F*-value	*P*-value	*F*-value	*P*-value	*F*-value	*P*-value	*F*-value	*P*-value	*F*-value	*P*-value	*F*-value	*P*-value
-2.00	0.43	0.65	1.88	0.15	0.56	0.57	0.11	0.89	3.66	0.03*	1.53	0.22
-1.50	0.45	0.63	0.49	0.61	0.12	0.88	1.01	0.36	3.94	0.023*	0.18	0.83
-1.00	2.25	0.11	1.31	0.27	2.32	0.10	4.73	0.011*	6.92	0.002*	1.17	0.31
-0.50	1.92	0.15	2.57	0.08	2.97	0.06	4.96	0.009*	7.01	0.001*	2.13	0.12
0.00	4.48	0.01*	5.36	0.006*	3.92	0.022*	6.32	0.003*	4.61	0.013*	0.42	0.65
0.50	1.66	0.19	1.36	0.25	2.23	0.11	1.15	0.31	2.37	0.09	2.51	0.08
1.00	0.13	0.87	0.71	0.49	0.09	0.91	1.65	0.19	0.23	0.79	1.09	0.33
1.50	0.37	0.69	0.55	0.57	0.06	0.93	0.49	0.61	0.77	0.46	0.53	0.59
2.00	0.96	0.38	0.27	0.75	0.11	0.89	1.23	0.29	0.15	0.86	0.05	0.94

Frequency analysis of treatment needs revealed a clear hierarchy in perception among the three groups. Orthodontists consistently demonstrated the highest frequency of recommending treatment across all midline deviations and conditions, with approval rates often exceeding 90%, particularly for major deviations (1.5 mm, 2.0 mm) and conditions, such as chin deviation (100% at 2 mm). General dentists showed an intermediate level of concern, while laypersons consistently reported the lowest frequency of treatment need, with *No* responses frequently reaching 40%-60% for minor deviations. A notable trend is that for the 0.0 mm (ideal) midline, the consensus broke down, with all groups showing nearly a 50/50 split, indicating a significant subjective interpretation of *normal*. As deviations increased, the frequency of *Yes* responses generally increased across all groups, but the gap between orthodontists and laypersons remained pronounced. The key inference is that professional training significantly increases the perception of treatment needs, with orthodontists being the most interventionist. Furthermore, conditions such as chin deviation and asymmetric upper lip elevation elicited higher treatment consensus than more subtle issues such as decreased upper incisor show (Table 3).

**Table 3:** Frequency distribution of treatment need assessment for altered smile conditions at different midline deviations, as evaluated by general dentists, laypersons, and orthodontists. Data are presented as frequency (*N*) and percentage (%), where *N* denotes the number of evaluators.

Deviation of upper midline with respect to facial midline (mm)	Evaluators	Category	Series A: Normal upper teeth show		Series B: Reduced upper teeth show		Series C: Increased gumminess		Series D: Maxillary cant		Series E: Asymmetric upper lip elevation		Series F: Chin deviation	
			N	%	N	%	N	%	N	%	N	%	N	%
-2	General dentists	No	8	26.67	10	33.33	8	26.67	7	23.33	3	10.00	5	16.67
		Yes	22	73.33	20	66.67	22	73.33	23	76.67	27	90.00	25	83.33
	Laypersons	No	14	46.67	15	50.00	10	33.33	9	30.00	12	40.00	11	36.67
		Yes	16	53.33	15	50.00	20	66.67	21	70.00	18	60.00	19	63.33
	Orthodontists	No	6	20.00	2	6.67	1	3.33	3	10.00	1	3.33	0	0.00
		Yes	24	80.00	28	93.33	29	96.67	27	90.00	29	96.67	30	100.00
-1.5	General dentists	No	12	40.00	7	23.33	6	20.00	11	36.67	5	16.67	5	16.67
		Yes	18	60.00	23	76.67	24	80.00	19	63.33	25	83.33	25	83.33
	Laypersons	No	11	36.67	11	36.67	13	43.33	10	33.33	11	36.67	9	30.00
		Yes	19	63.33	19	63.33	17	56.67	20	66.67	19	63.33	21	70.00
	Orthodontists	No	7	23.33	5	16.67	2	6.67	5	16.67	3	10.00	2	6.67
		Yes	23	76.67	25	83.33	28	93.33	25	83.33	27	90.00	28	93.33
-1	General dentists	No	11	36.67	9	30.00	11	36.67	10	33.33	8	26.67	8	26.67
		Yes	19	63.33	21	70.00	19	63.33	20	66.67	22	73.33	22	73.33
	Laypersons	No	14	46.67	13	43.33	13	43.33	16	53.33	13	43.33	12	40.00
		Yes	16	53.33	17	56.67	17	56.67	14	46.67	17	56.67	18	60.00
	Orthodontists	No	6	20.00	4	13.33	5	16.67	8	26.67	6	20.00	3	10.00
		Yes	24	80.00	26	86.67	25	83.33	22	73.33	24	80.00	27	90.00
-0.5	General dentists	No	15	50.00	15	50.00	11	36.67	10	33.33	7	23.33	8	26.67
		Yes	15	50.00	15	50.00	19	63.33	20	66.67	23	76.67	22	73.33
	Laypersons	No	20	66.67	20	66.67	12	40.00	18	60.00	14	46.67	15	50.00
		Yes	10	33.33	10	33.33	18	60.00	12	40.00	16	53.33	15	50.00
	Orthodontists	No	13	43.33	11	36.67	14	46.67	11	36.67	7	23.33	5	16.67
		Yes	17	56.67	19	63.33	16	53.33	19	63.33	23	76.67	25	83.33
0	General dentists	No	14	46.67	19	63.33	13	43.33	15	50.00	11	36.67	13	43.33
		Yes	16	53.33	11	36.67	17	56.67	15	50.00	19	63.33	17	56.67
	Laypersons	No	16	53.33	17	56.67	16	53.33	15	50.00	19	63.33	18	60.00
		Yes	14	46.67	13	43.33	14	46.67	15	50.00	11	36.67	12	40.00
	Orthodontists	No	15	50.00	15	50.00	9	30.00	16	53.33	11	36.67	12	40.00
		Yes	15	50.00	15	50.00	21	70.00	14	46.67	19	63.33	18	60.00
0.5	General dentists	No	7	23.33	8	26.67	7	23.33	4	13.33	12	40.00	7	23.33
		Yes	23	76.67	22	73.33	23	76.67	26	86.67	18	60.00	23	76.67
	Laypersons	No	13	43.33	13	43.33	11	36.67	9	30.00	11	36.67	16	53.33
		Yes	17	56.67	17	56.67	19	63.33	21	70.00	19	63.33	14	46.67
	Orthodontists	No	6	20.00	6	20.00	8	26.67	6	20.00	8	26.67	4	13.33
		Yes	24	80.00	24	80.00	22	73.33	24	80.00	22	73.33	26	86.67
1	General dentists	No	8	26.67	10	33.33	6	20.00	13	43.33	9	30.00	4	13.33
		Yes	22	73.33	20	66.67	24	80.00	17	56.67	21	70.00	26	86.67
	Laypersons	No	13	43.33	14	46.67	10	33.33	13	43.33	8	26.67	12	40.00
		Yes	17	56.67	16	53.33	20	66.67	17	56.67	22	73.33	18	60.00
	Orthodontists	No	3	10.00	4	13.33	4	13.33	6	20.00	2	6.67	3	10.00
		Yes	27	90.00	26	86.67	26	86.67	24	80.00	28	93.33	27	90.00
1.5	General dentists	No	9	30.00	5	16.67	5	16.67	7	23.33	5	16.67	6	20.00
		Yes	21	70.00	25	83.33	25	83.33	23	76.67	25	83.33	24	80.00
	Laypersons	No	10	33.33	10	33.33	13	43.33	13	43.33	9	30.00	7	23.33
		Yes	20	66.67	20	66.67	17	56.67	17	56.67	21	70.00	23	76.67
	Orthodontists	No	2	6.67	1	3.33	1	3.33	2	6.67	1	3.33	2	6.67
		Yes	28	93.33	29	96.67	29	96.67	28	93.33	29	96.67	28	93.33
2	General dentists	No	7	23.33	7	23.33	3	10.00	11	36.67	5	16.67	4	13.33
		Yes	23	76.67	23	76.67	27	90.00	19	63.33	25	83.33	26	86.67
	Laypersons	No	10	33.33	10	33.33	10	33.33	12	40.00	9	30.00	6	20.00
		Yes	20	66.67	20	66.67	20	66.67	18	60.00	21	70.00	24	80.00
	Orthodontists	No	3	10.00	1	3.33	1	3.33	3	10.00	1	3.33	0	0.00
		Yes	27	90.00	29	96.67	29	96.67	27	90.00	29	96.67	30	100.00

The chi-square test results revealed that a statistically significant association (*P* < 0.05) between evaluators and their perception of treatment need was not consistent but was highly dependent on the magnitude of midline deviation. Significant associations were concentrated at the extremes of the spectrum for major deviations (1.0-2.0 mm) and, notably, for the most negative deviation (-2.0 mm). At the ideal midline (0.0 mm), no significant associations were found for any condition, indicating that a lack of consensus was random and not systematically different between the groups. The conditions that most frequently showed significant differences were decreased upper teeth show, asymmetric upper lip elevation, and chin deviation. The inference is that group affiliation is a significant determinant of treatment perception, primarily for more pronounced, clinically relevant asymmetries, while perceptions of a perfectly aligned or very subtly deviated midline are more uniform and subjective across all groups (Table 4).

**Table 4:** Association between groups and treatment need for altered smile conditions at different midline deviations. Values represent chi-square statistics with corresponding *P*-values. *P < 0.05 denotes statistical significance using the chi-square test.

Deviation of upper midline with respect to facial midline (mm)	Series A: Normal upper teeth show		Series B: Reduced upper teeth show		Series C: Increased gumminess		Series D: Maxillary cant		Series E: Asymmetric upper lip elevation		Series F: Chin deviation	
	Chi stats	*P*-value	Chi stats	*P*-value	Chi stats	*P*-value	Chi stats	*P*-value	Chi stats	*P*-value	Chi stats	*P*-value
-2.00	5.39	0.06	13.65	0.01*	8.94	0.01*	3.75	0.15	15.65	0.01*	13.84	0.01*
-1.50	2.11	0.35	3.27	0.19	11.55	0.01*	3.35	0.18	6.93	0.03*	5.62	0.06
-1.00	4.82	0.09	6.59	0.03*	5.29	0.07	4.91	0.08	4.12	0.12	7.12	0.01*
-0.50	3.48	0.17	5.42	0.06	0.64	0.72	5.15	0.07	5.08	0.07	8.19	0.01*
0.00	0.26	0.87	1.08	0.58	3.37	0.18	0.08	0.95	7.49	0.11	2.76	0.25
0.50	4.61	0.09	4.12	0.12	1.41	0.49	2.53	0.28	1.27	0.52	12.38	0.01*
1.00	8.52	0.01*	7.88	0.01*	3.61	0.16	4.75	0.09	5.73	0.05	9.74	0.01*
1.50	7.08	0.02*	9.27	0.01*	14.94	0.01*	10.94	0.01*	7.68	0.01*	3.36	0.18
2.00	4.75	0.09	8,75	0.01*	11.33	0.01*	7.89	0.01*	7.68	0.01*	6.31	0.04*

## Discussion

The observed variations in perceptual thresholds among orthodontists, general dentists, and laypersons underscore the importance of professional expertise in evaluating smile esthetics. Orthodontists exhibited heightened sensitivity to minor upper midline discrepancies, particularly within -1.0 to 0.0 mm, likely due to their specialized training in orthodontics, which emphasizes precise alignment and symmetry. This aligns with prior research indicating that orthodontic professionals detect deviations as small as 1-2 mm, whereas laypeople often require larger shifts, such as 3-4 mm, before noticing esthetic impairments [12-15]. Ferreira et al. [12] reported that laypersons were capable of perceiving deviations in the upper midline when such deviations exceeded 2 mm, particularly when the smile incorporated the lip profile, as observed in our investigation. Kokich et al. [13] demonstrated that orthodontists identified midline shifts of 4 mm as unacceptable, whereas laypersons and general dentists were unable to perceive a midline deviation of 4 mm.

Similarly, Johnston et al. [14] found that dental professionals' rigorous exposure to ideal esthetic parameters sharpens their discernment, leading to a lower tolerance for asymmetries compared to non-professionals. They reported that laypersons were unable to detect an upper midline deviation of less than 2 mm. This professional-layperson gap may stem from orthodontists' familiarity with cephalometric norms and smile arc principles, fostering a bias toward intervention for subtle deviations that might enhance overall facial harmony. Tahir et al. [15] found that laypersons could not perceive upper midline deviations up to 4 mm, whereas dental professionals were able to perceive deviations above 2 mm.

In the context of gingival display modifications, the interplay between midline discrepancies further amplifies the perceptual differences. Increased gingival exposure (up to 3 mm) compounded the unattractiveness of midline shifts, especially among professionals, reflecting established esthetic guidelines that deem excessive gummy smiles as detracting from smile appeal [7]. A study by Negruțiu et al. [16] showed that gingival displays of 1.43 ± 3.785 mm were evaluated least attractive across evaluators, and −0.57 ± 2.407 mm as the most attractive. This could be linked to evolutionary preferences for balanced facial proportions, where excessive gum disrupts the tooth-lip relationship, evoking perceptions of immaturity or disproportion. Conversely, reduced upper teeth show mitigated midline effects, possibly because it minimizes visual emphasis on the dentition, allowing evaluators to focus on broader facial cues. This is supported by a systematic review by Batra et al. [17], which indicated that laypersons prioritize overall facial attractiveness over isolated dental features, tolerating gingival variations unless they exceed 2.7 mm. Cultural factors may also influence these perceptions. In Asian populations, similar to the Indian cohort, moderate gingival display is often viewed more favorably than in Western contexts, as noted in cross-cultural comparisons by Nomura et al. [18], potentially explaining the convergence in ratings for pronounced deviations.

Chin deviation introduces an additional layer of complexity that interacts with midline discrepancies to heighten treatment recommendations, particularly among orthodontists. A 2 mm chin shift to the right or left exacerbated the perceived asymmetry, aligning with the findings that facial skeletal deviations amplify dental esthetic judgments [9,19]. Silva et al. [19] reported that chin asymmetries of 3°-4° are detectable by professionals but often overlooked by laypersons until reaching 5°-6°, attributing this to orthodontists' holistic evaluation of facial midlines, including skeletal components. The underlying reason may involve visual processing biases, where the chin serves as a reference point for facial symmetry, and deviations disrupt the general perception of the face. Kuruhan et al. [20] reported that chin deviation to the same side as upper midline deviation was more acceptable and less perceivable by both orthodontists and laypersons than chin deviation to the opposite side. This could have been because same-side chin and upper midline deviations are more acceptable due to visual coherence, aligning with natural asymmetries and reducing perceived disruption compared to opposite-side deviations [21]. Orthodontists advocate treatment more frequently, viewing chin-midline interactions as indicators of underlying malocclusion or skeletal disharmony that require interdisciplinary intervention.

The 5° maxillary cant, with the right side shifted downward and the left side elevated, significantly influenced perceptions, particularly among orthodontists, who rated these smiles as less attractive than general dentists and laypeople. This can be explained by the disruption of the occlusal plane, which orthodontists are trained to assess as a critical component of smile aesthetics. A canted maxilla creates an uneven smile line, misaligning the teeth relative to the facial midline and lip curvature, which professionals perceive as a functional and esthetic concern. Kaya and Uyar [22] reported that maxillary canting, along with increased gingival display, negatively influences smile attractiveness, associating them with underlying skeletal asymmetries requiring correction. However, laypeople may overlook such deviations unless they exceed 4°, as their judgments prioritize overall facial harmony over specific dental alignments [23]. The reason for this discrepancy lies in the orthodontists’ analytical approach, which focuses on occlusal plane deviations as indicators of potential malocclusion or skeletal issues, whereas laypeople rely on holistic impressions and tolerate minor cants if the smile remains visually cohesive.

The 2 mm elevation of the right commissure in asymmetric upper lip elevation further amplified perceptual differences, with orthodontists again showing the highest sensitivity. This deviation disrupts the parallelism of the lower lip line with the occlusal plane, creating a visually striking asymmetry that professionals associate with dynamic smiling. Mathis et al. [24] found that laypersons notice upper lip asymmetries of 2.5 mm or greater as they alter the smile’s dynamic balance. The heightened professional sensitivity may stem from an understanding of the neuromuscular factors influencing lip movement, which could indicate underlying issues, such as facial nerve dysfunction or dental misalignment. These perceptual disparities can be attributed to differences in cognitive frameworks. Professionals apply analytical, detail-oriented assessments supported by education and clinical experience, while laypersons rely on holistic, emotional responses influenced by media and personal experiences. In support of this, Pinho et al. [25] found that laypersons' judgments were swayed by overall attractiveness rather than specific metrics, leading to greater tolerance.

Clinical implications

These insights guide orthodontic practice by highlighting the need for patient-centered communication, especially when addressing minor deviations that professionals deem critical but patients may not notice. Clinicians should use digital simulations to align expectations, potentially reducing overtreatment in cases in which lay perceptions dominate satisfaction. For combined discrepancies, prioritizing skeletal corrections, such as chin deviations, could optimize outcomes by integrating multidisciplinary approaches for enhanced esthetics.

Limitations

The study's Indian setting introduces cultural bias, which potentially does not reflect global views, thus limiting the generalizability of the study. Posed smiles may not capture dynamic expressions, and the subjectivity of VAS scoring could introduce variability despite its high reliability. The use of one-way ANOVA without post-hoc testing limits the granularity of intergroup comparisons, and a two-way or mixed ANOVA could have better captured interactions between evaluator groups and esthetic parameters. Future studies should employ these methods to enhance analytical robustness. Future studies should also incorporate longitudinal assessments and a broader demographic population.

## Conclusions

This study revealed that orthodontists exhibit significantly higher sensitivity to minor upper midline discrepancies, maxillary cant, and asymmetric upper lip elevation than general dentists and laypersons. Increased gingival display and chin deviation amplified the perceived need for correction across all groups, with orthodontists consistently advocating interventions at higher rates, particularly for major deviations. Same-side chin and midline deviations were less noticeable than opposite-side deviations, likely because of visual coherence. These findings highlight the influence of professional training on esthetic perception and underscore the need for tailored patient communication to align clinical recommendations with patient expectations, particularly for subtle asymmetries that laypersons may not perceive.

## Figures and Tables

**Figure 2:**
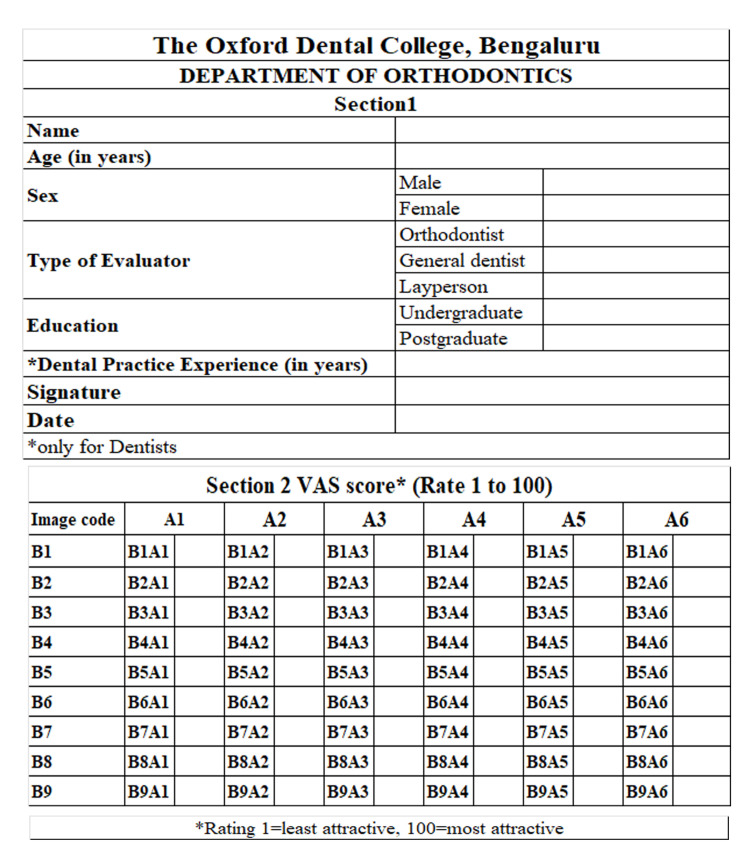
Original questionnaire form used in the study. Credit: Khadeer Riyaz.

## Appendices

Appendix 
